# Causes and consequences of purifying selection on SARS-CoV-2

**DOI:** 10.1093/gbe/evab196

**Published:** 2021-08-24

**Authors:** Atahualpa Castillo Morales, Alan M Rice, Alexander T Ho, Christine Mordstein, Stefanie Mühlhausen, Samir Watson, Laura Cano, Bethan Young, Grzegorz Kudla, Laurence D Hurst

**Affiliations:** 1 The Milner Centre for Evolution, Department of Biology and Biochemistry, University of Bath, Bath, BA2 7AY, UK; 2 MRC Human Genetics Unit, Institute for Genetics and Molecular Medicine, The University of Edinburgh, Edinburgh, UK; 3 Department of Molecular Biology and Genetics, Aarhus University, C F Møllers Allé 3, Aarhus, 8000, Denmark

**Keywords:** SARS-CoV-2, mutation rate, purifying selection, codon usage

## Abstract

Owing to a lag between a deleterious mutation’s appearance and its selective removal, gold-standard methods for mutation rate estimation assume no meaningful loss of mutations between parents and offspring. Indeed, from analysis of closely related lineages, in SARS-CoV-2 the Ka/Ks ratio was previously estimated as 1.008, suggesting no within-host selection. By contrast, we find a higher number of observed SNPs at 4-fold degenerate sites than elsewhere and, allowing for the virus’s complex mutational and compositional biases, estimate that the mutation rate is at least 49-67% higher than would be estimated based on the rate of appearance of variants in sampled genomes. Given the high Ka/Ks one might assume that the majority of such intra-host selection is the purging of nonsense mutations. However, we estimate that selection against nonsense mutations accounts for only ∼10% of all the “missing” mutations. Instead, classical protein-level selective filters (against chemically disparate amino acids and those predicted to disrupt protein functionality) account for many missing mutations. It is less obvious why for an intracellular parasite, amino acid cost parameters, notably amino acid decay rate, are also significant. Perhaps most surprisingly, we also find evidence for real time selection against synonymous mutations that move codon usage away from that of humans. We conclude that there is common intra-host selection on SARS-CoV-2 that acts on nonsense, missense and possibly synonymous mutations. This has implications for methods of mutation rate estimation, for determining times to common ancestry and the potential for intra-host evolution including vaccine escape.

**Significance statement:**

In SARS-CoV-2 we find evidence for common intra-host purifying selection against nonsense, missense and synonymous mutations, such that the true underlying mutation rate is about 50% higher than would be estimated if one assumes that the mutation rate is the rate of appearance of mutations in the circulating population. This has implications for methods to determine mutation rates, for determining times to common ancestry and the potential for vaccine escape.

## Introduction

Classically purifying selection can be inferred by absence. For example, in the Ka/Ks test we employ the normalised rate of occurrence of substitutions at synonymous sites (Ks) in a protein coding gene as a measure of the background rate of evolution, comparing this to the normalised rate of non-synonymous changes (Li, et al. 1985; Goldman and Yang 1994). A dearth of the latter compared to the former (Ka/Ks<1) is taken to imply that protein changing mutations happened but were too deleterious to persist (Li, et al. 1985; Goldman and Yang 1994). The method thus implicitly infers the rate of what might be called "missing" mutations.

A consequence of this is that, owing to a lag between mutation appearance and selective removal (Rocha, et al. 2006), our ability to resolve purifying selection on recently diverged lineages is weak, few mutations being "missing" (Ponting 2008). Indeed, for this reason, for closely related species Ka/Ks in a pairwise analysis declines as the time to common ancestry increases (Rocha, et al. 2006). Consequently, we know relatively little about the activity of purifying selection over the short term (Ponting 2008), let alone what might be called "real time". Similarly, to estimate the mutation rate (meaning the rate at which new mutations happen, not the rate of lineage evolution) we employ a few generations of mutation accumulation lines (Lynch, et al. 2016) under the assumption that the rate of accumulation of changes in DNA/RNA is the mutation rate, as purifying selection is both diminished and will not yet have influenced the fate of mutations. Indeed, parent-offspring trios are now considered a gold standard for mutation rate estimation as such analyses are presumed to be the least affected by the missing mutation problem (Yang, et al. 2015).

An ideal examination of real time selection in the wild would require analysis of massive numbers of full genomes of a relatively fast evolving species sampled continuously in time and place. Such a natural experiment is currently running. Indeed, the volume of genome data for SARS-CoV-2 allows an unparalleled evaluation of the activity of purifying selection in real time. Early analysis, however, suggested that purifying selection was not detectable, Ka/Ks being almost exactly 1 (Bai, et al. 2020) i.e. there is no distortion from the immediate mutational profile, consistent with assumptions of parent-offspring mutation rate estimation. More recent evidence, by contrast, indicates that such selection is detectable (Dearlove, et al. 2020; Shen, et al. 2020; Tang, et al. 2020; Tonkin-Hill, et al. 2020; Lythgoe, et al. 2021). Similarly, mutational scanning experiments indicate positions under positive and negative selective constraints in the SARS-CoV-2 receptor binding domain (Starr, et al. 2020).

There are numerous reasons why the study of real time purifying selection in SARS-CoV-2 in particular might be interesting. For example, the difference between the rate of appearance of new mutations in the population and the rate at which they actually occur, is indicative of the potential for intra-host evolution. If, for example, there is little disparity (e.g. Ka/Ks=1) then intra-host selection is not occurring and the nonsynonymous mutations that occur are being transmitted without selection. Conversely, if only a small proportion of actual mutations survive to be transmitted, the adaptive potential, for example for selection for vaccine escape, must be quite high, there being differential birth and death (i.e. intra-host variance in fitness with the viral clone). Similarly, if we infer the evolutionary rate of a virus by assaying the rate at which RNA changes appear in the population (Duchene, et al. 2020; Hill and Rambaut 2020; Nextstrain 2020) and, in turn, assume this to reflect the true underlying rate (much as done with parent-offspring sequencing), then the true underlying rate is likely to be under-estimated. While not necessarily important for inferring the evolutionary rate, allowance for such purifying selection can affect estimation of time to common ancestry (Wertheim and Kosakovsky Pond 2011). Here then we attempt to estimate the proportion of mutations that occurred but were missing prior to sequencing of circulating variants. From this we attempt in turn to infer the true mutation rate, more particularly asking whether this is a sizeable correction or not. That Ka/Ks ~1, might suggest that no meaningful correction is needed.

Further, the profile of these missing mutations may also contain information as to what selection is acting on. Selection against most nonsense mutations seems inevitable. Indeed, it is possible both that there is purifying selection operating against nonsense mutations and that Ka/Ks=1, as the later metric does not factor in nonsense mutations. We should then predict fewer nonsense mutations circulating within the sequenced genomes than expected given the underlying mutational profile. Prior sampling of intra-individual variation supports this (Tonkin-Hill, et al. 2020), although sequence quality issues may be relevant here (see Nekrutenko 2020). Indeed, for reasons unknown (see Nekrutenko 2020), one commonly employed intra-host sequencing project (SRP253798) reports both remarkably high numbers of mutations and that almost all such mutations are C->U. This has the potential to over-estimate the rate of generation of nonsense mutations. Given that Ka/Ks (that considers only missense/nonsynonymous changes) is near unity (Bai, et al. 2020), one might then suggest that, despite evidence for purifying selection against some missense (nonsynonymous) variants (Dearlove, et al. 2020; Lythgoe, et al. 2021), the vast majority of purifying selection must be against nonsense mutations. Here we attempt to assay whether this is so.

We find that there is common purifying selection operating at the protein level (i.e. against nonsynonymous variants). We then ask whether the profile of selection against non-synonymous variation seen in more distant comparisons can be detected in real time. Classically nonsynonymous mutations are selected against when they disrupt protein function too much. This can be reflected in a dearth of fixed (between two different species) differences that see an amino acid replaced by one that is chemically very different (Weber and Whelan 2019). We ask whether we can detect such selection operating within hosts. In addition, we might expect, at a higher level of granularity, that a biophysical model of protein functioning might predict which amino acid exchanges are tolerated. We consider spike protein as an exemplar, not least because the model for this protein was not informed by evolutionary constraint data (which would render any analysis circular).

Analyses of longer-term purifying selection suggests that mutations to more biosynthetically costly amino acids are also subject to purifying selection (Richmond 1970; Akashi and Gojobori 2002; Heizer, et al. 2006; Hurst, et al. 2006; Swire 2007; Charneski, et al. 2011). In contrast to the above predictors, we don't necessarily expect this to be detectable, in real time or otherwise, in a virus which may itself not suffer the costs of amino acid synthesis, the ATP costs of amino acid biosynthesis being more obviously suffered by the host not the virus. One might, however, conjecture that what is good for the host might also be good for the virus (fitness covariance) and, as translation imposes the majority of the cost of building a virus, such costs may be under selection (Mahmoudabadi, et al. 2017). Indeed, virus-like Gene Transfer Elements integrated in *Alphaproteobacteria* have been suggested to be under positive selection for the reduction of cost (Kogay, et al. 2020). However, an integrated element is expected to have stronger fitness covariance with its host than SARS-CoV-2 for whom the host is just a temporary transmission vehicle.

Perhaps the weakest selection we might hope to detect is that of synonymous mutations. Although selection on synonymous sites is likely to be hard to detect, prior evidence suggests viruses might adapt their codon usage to that of their host (Hernandez-Alias, et al. 2021), to optimize translational efficiency (Wong, et al. 2010; Liu, et al. 2011; Fan, et al. 2015; Chen, et al. 2020;

Hernandez-Alias, et al. 2021) or avoid certain nucleotide combinations (Shpaer and Mullins 1990; Atkinson, et al. 2014; Gaunt, et al. 2016; Gu, et al. 2019). Some evidence for selection of codon usage in SARS-CoV-2 has been reported (Gu, et al. 2020; De Maio, et al. 2021; Hernandez-Alias, et al. 2021). Our prior analysis reveals that predicted neutral mutational equilibrium content of U at 4 fold degenerate sites (U4*) at 65% is higher than the observed U4, which could indicate purifying selection on U mutations at 4-fold degenerate sites but could also reflect a relatively recent change in mutational profile and lag to mutational equilibrium (Rice, et al. 2020).

Here then, in addition to estimating the number of missing mutations, we examine nonsense, missense and synonymous mutations to test particular hypotheses for the causes of such selection. Although the genomic resources are exceptional, SARS-CoV-2 analysis presents unusual methodological challenges. Site frequency spectra (SFS) approaches have been applied in an attempt to infer selection on nucleotide composition in SARS-CoV-2 (e.g. De Maio, et al. 2021). However, broader application of such methods may well be problematic as some methods are advised against in non-recombining genomes (Bustamante, et al. 2001) and inferences can be confounded by effects of demography that can mimic selection. Indeed, SFS methods are more commonly employed to determine demography (Lapierre, et al. 2016), analyses that in turn are confounded by their failure to allow for weak selection (Lapierre, et al. 2016). Moreover, highly geographically skewed sequencing efforts, including intensive sequencing around outbreak hotspots, will distort the SFS (for example, a rare mutation in an over-sequenced location will appear to be at a relative high net frequency).

Ka/Ks has also been applied to test for selection on SARS-CoV-2 (see e.g. Bai, et al. 2020). Aside from the fact that the test was designed to be applied to fixed between-species differences (Mugal, et al. 2020), this test too has numerous interrelated issues. First, it overlooks nonsense mutations as a source of “missing” mutations. Second, even the best codon centred models (Goldman and Yang 1994; Wertheim and Kosakovsky Pond 2011) ignore complex mutational effects that bridge between codons, forcing codon pair bias, that is important for viral functioning (Coleman, et al. 2008). Third, and related, SARS-Cov-2 has an exceptionally biased and complex mutational profile (Rice, et al. 2020; Simmonds 2020b; Graudenzi, et al. 2021), with a large bias towards U, especially from CU and GU dinucleotides, that is likely to confound estimation methods. Coupled with differential nucleotide usage at different codon positions, this is likely to interfere with estimation. For example, while one could estimate the true mutation rate by using the rate at four-fold degenerate codon sites alone (cf Keightley and Eyre-Walker 2000), as these are much more U

biased than the other codon sites (Rice, et al. 2020), the rate at four fold degenerate sites will not reflect the underlying rate at the other sites, potentially underestimating it as U has a low mutation rate (Rice, et al. 2020). Compounded with a short time between mutational occurrence and sampling, these issues may explain why prior Ka/Ks estimation reports a value of 1.008, indicative of no purifying selection (Bai, et al. 2020).

To overcome these problems, we apply a variety of methods. Most notably we estimate rates at four-fold sites of different nucleotide compositions and use these nucleotide-dependent rates to infer the true underlying mutation rate and hence the rate of missing mutations, given the nucleotide content of all other sites. Similarly, to determine the profile of missing mutations we define expectations of the rates of amino acids exchanges under a complex null neutral model and examine the predictors of deviations from this. Using related methods we also attempt to infer the direction of selection on synonymous mutations. These methods have an advantage over direct within-host sampling that they can also estimate rate of mutations so deleterious that they never attain reasonable frequencies within the host. They should also be less subject to sequencing artefact known to affect intra-host sampling (see Nekrutenko 2020). We also however employ such within individual sequencing to infer selection.

## Results

### An excess of variants at four-fold degenerate sites implies purifying selection

Were selection ongoing we expect that, per occurrence of a given nucleotide, the number of mutations observed at 4-fold degenerate sites would be higher than at sites 1 and 2 in codons. In all eight independent comparisons (4-fold site v site 1, 4-fold site v site 2, for 4 nucleotides), the 4-fold degenerate sites have more mutations per occurrence of the ancestral nucleotide (Fig 1a: binomial test, P=0.008). This is consistent with weaker selective constraint on mutations at 4-fold sites detectable even at sites recently sampled (Fig 1a). We also see that when all 12 mutational types are considered, 4-fold degenerate sites have the highest rate in 22/24 comparisons (Fig 1b: binomial test, P=3.6 x $10^{-5}$).

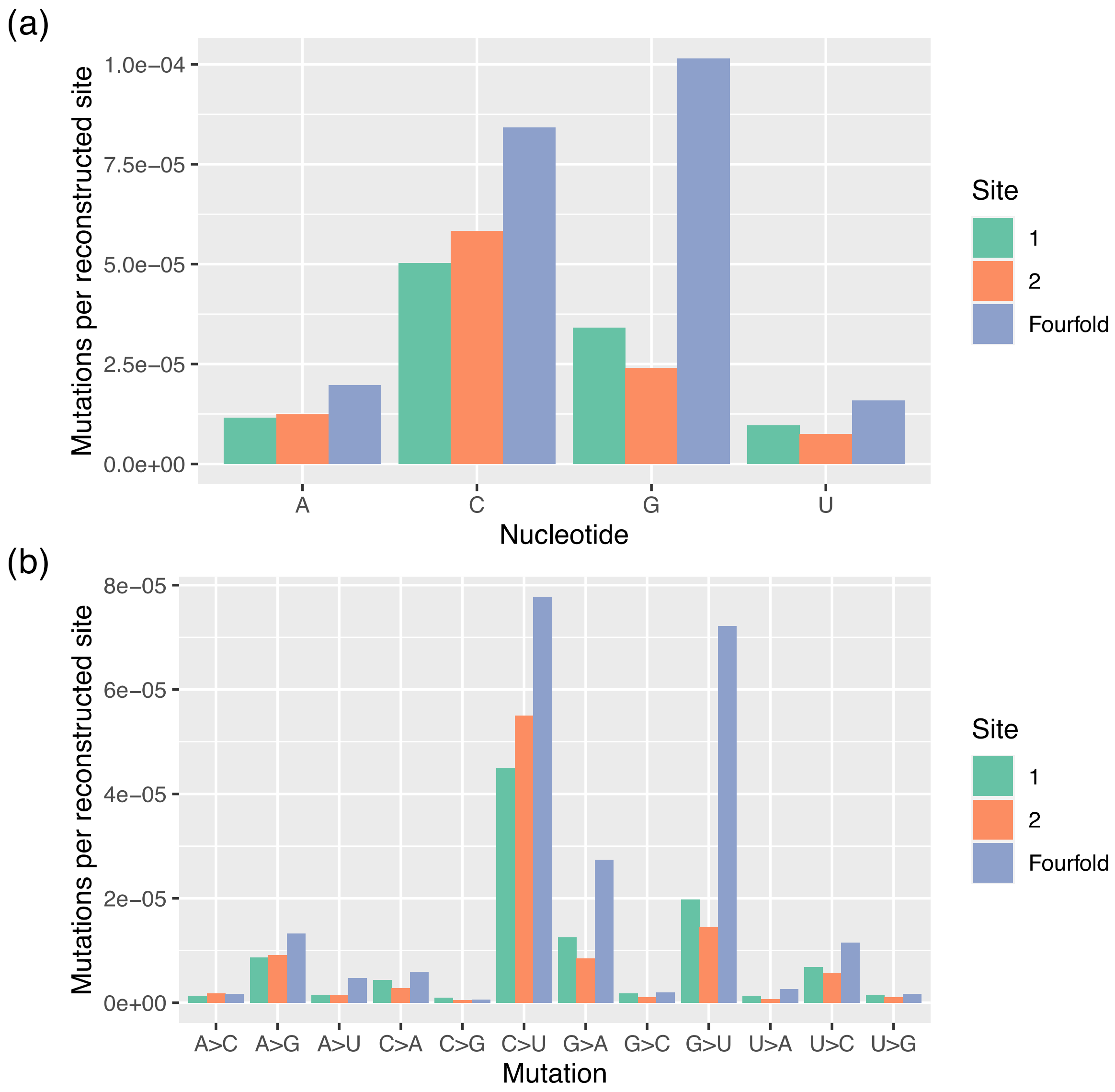

**Figure 1. Comparisons between fourfold and codon site 1 and site 2 mutations. A.** Rate of observed mutation per reconstructed (ie alignable and qualifying) site in the genome for each base (pre mutation). **B.** The same data as in figure A divided by type of mutation given ancestral state. When all 12 mutational types are considered, 4-fold degenerate sites have the highest rate in 22/24 comparisons (binomial test, P=3.6 x $10^{-5}$).

To allow for dinucleotide effects, not considered when performing standard Ka/Ks tests, as performed for SARS-CoV-2 (see e.g. Lythgoe, et al. 2021), we also consider the incidence rate of mutations centred on a given base at a 4-fold degenerate site in each of the 16 possible dinucleotides (either at sites 2 and 3, denoted “23”, or 3 and 1, “31”) and compare this with observations for the same dinucleotides where the mutations observed are not centred over codon 3rd sites. The finding of a weaker selective constraint at four-fold degenerate sites is resilient to such control (Fig 2). All four nucleotides are more mutable when situated at a fourfold position, regardless of dinucleotide (Wilcoxon ranked-sum tests; A: $p = 0.0052$, C: $p = 9.8 \times 10^{-5}$, G: $p = 0.00021$, U: $p = 0.00024$).

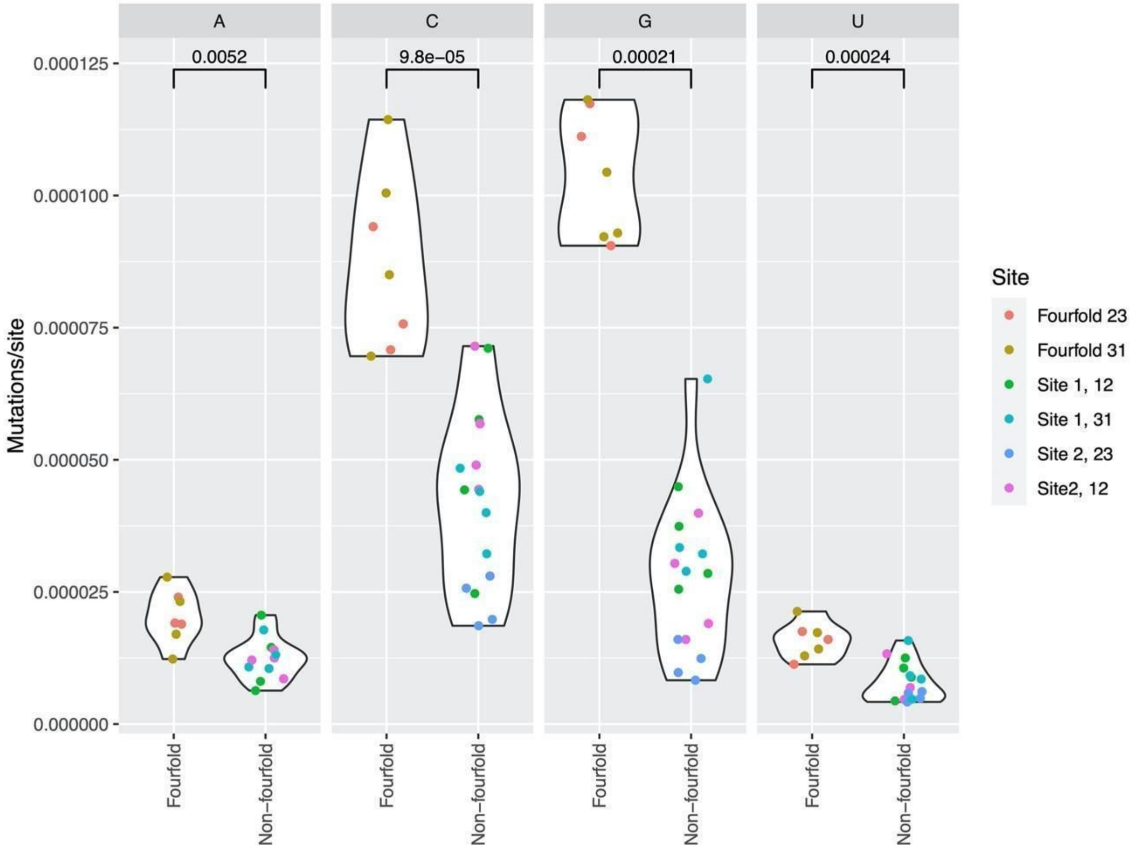

**Figure 2. Comparisons between fourfold and non-fourfold mutations at different reconstructed dinucleotide sites.** The increased mutability of fourfold sites is resilient to control for dinucleotide effects.

**For every ten variants we see, around 5 other mutations are not recovered**

The above evidence indicates that there must be some missing mutations derived from codon sites 1 and 2. If $x$ is the number of new mutations seen per unit time down a particular lineage then $x + dx$ must be the true rate, $dx$ being the mutations that happened but disappeared before they were sequenced. How can we estimate $dx$ and hence the true mutation rate, $x + dx$? Under the assumption of no selection on 4-fold degenerate sites, and assuming that most mutations are either neutral or deleterious, then the difference between their rate and that observed elsewhere in the genome (Fig 1) is informing us of the rate of missing mutations. One could, alternatively, estimate the rate at 4-fold sites and assume all other sites have the same rate. However, we have previously identified both strong nucleotide skews at 4-fold sites and strong biases in the mutation rate per occurrence of each of the nucleotides (Rice, et al. 2020). Considering that codon sites 1 and 2 are not as skewed in nucleotide content as 4-fold sites (Rice, et al. 2020), the optimum approach is to extrapolate from the patterns at 4-fold sites in a manner that is sensitive to differences in nucleotide composition across sites.

*dx* can be estimated as the number of mutations seen in sequencing data multiplied by the proportion of mutations missing ($P_m$) (this being the proportion in terms of those observed), which may be estimated by comparing rates at codon sites 1, 2, and 3 to those at 4-folds (see methods for calculation). As dx = x. $P_m$, the true mutation rate = x [1+ $P_m$] per unit time. We estimate $P_m$=0.672 i.e., we are seeing 1/1.672 = 59.8% of all mutations, missing 40.2% and the true mutation rate is 1.672 times higher than that observed. Most of the mutations missing are at G nucleotides. At A sites, we are seeing 70.0% of mutations and missing 30.0%, this equating to 3,119 mutations lost in the analysed phylogeny. At C sites we are missing 22.4% (5,735 mutations), at G sites we are missing 61.5% (20,974 mutations), and at U sites we are missing 21.8% (1,969 mutations) of mutations. In total we estimate there are 31,797 unsequenced mutations missing in total.

Using mutational counts at the dinucleotide level, we may also estimate $P_m$ and *dx* (and the number of mutations missing for each dinucleotide) by adapting the above method. For example, the mutation rate of A in an AG dinucleotide at site 12 may be compared to the mutation rate of A at AG dinucleotides where A is the fourfold site. The mutation rate of G in an AG dinucleotide at site 12 is compared to the mutation rate of G at AG dinucleotides where the G is the fourfold site, and so on. Owing to the structure of the genetic code, there are no fourfold sites following a second codon position A, hence for these dinucleotides we use the mutation rates at codon third sites, rather than fourfolds rates, for the comparison. The resulting predicted number of missing mutations is hence likely to be an underestimate. Nevertheless, from our dinucleotide calculations we estimate $P_m$=0.489 i.e., we are seeing 1/1.489 = 67.1% of all mutations, missing 32.9% and the true mutation rate is 1.489 times higher than that observed. In terms of raw mutations, this equates to 44,966 missing dinucleotide changes or 22,433 mutations (as each point mutation affects two dinucleotides). Given the probable underestimation, this corroborates the mononucleotide prediction of ~30,000 missing mutations. Indeed, consistent with most missing mutations being at G sites, our dinucleotide analysis predicts that mutations are most commonly missing from GG (9,963 mutations) and UG (8,867 mutations) dinucleotide sites.

Currently, the rate of SARS-CoV-2 sequence change is estimated from circulating mutations to be about 1 every two weeks or ~1 x $10^{-3}$ per site per year (Duchene, et al. 2020; Hill and Rambaut 2020; Nextstrain 2020). We hence suggest the mutation rate to be ~1.5-1.7 x $10^{-3}$ per site per year, assuming no selection at 4-fold degenerate sites.

**Selection skews the mutational matrix**

It is possible that purifying selection acts in a uniform fashion against all sites, in which case all mutations at second sites (none of which can be synonymous) will be equally under-represented when compared to 4-fold degenerate sites (N.B. a few C<->U (Leu<->Leu) and A<->G (Arg<->Arg) 1st site mutations are synonymous). This appears not to be the case with considerable heterogeneity between mutation types. Mutations from G are poorly tolerated at site 1 and 2 (Fig 1) and in particular G->U mutations appear to be commonly counter selected (we presume here that the 4-fold site rate does not indicate positive selection for U at such sites, not least because U4 observed (50.8%) is much less than neutral equilibrium predicted U4 content (65.6%) (Rice, et al. 2020).

To more systematically assess any such skew and the net effect on nucleotide composition, we compare the equilibrium nucleotide contents predicted on knowledge of the mutational profiles. We show using such a method that mutations at 4-fold degenerate sites and those not at 4-fold degenerate sites resulted in significantly different predicted mutational equilibria, with G under-represented at 4-fold sites (Z = -8.43), but still very rare, while U is very common but non-deviant between the two sets (Z = -0.35). To fully understand the variation between sites, we extend these calculations to consider sites 1, 2 and 4 folds separately. This reveals that all three classes of site within a codon are different from all others (Table 1). We conclude that selection not only prevents mutations at certain sites from increasing in frequency, but it also skews the mutational matrix with the nature of skew particular to the site concerned.

**Table 1. Comparisons between equilibrium vectors.** P is determined by 10,000 simulations (see methods). Z score orientation is such that a positive value implies comparative enrichment within the first comparator in the Comparison column. For example, in row 1 (4 vs non-4), the four fold degenerates sites are site class 1 and non-4 fold degenerate sites are the non-four fold degenerate sites (ie all others) and are class 2. In this case C* 1 for example is then the equilibrium

C content of sites of class 1 (4 fold degenerates) and C* 2 the equilibrium C content of sites of class 2.

| Comparisons | p-value | A* 1 | A* 2 | A: Z-score | C* 1 | C* 2 | C: Z-score | G* 1 | G* 2 | G: Z-score | U* 1 | U* 2 | U: Z-score |
|---|---|---|---|---|---|---|---|---|---|---|---|---|---|
| 4 vs non-4 | 0.012 | 0.170 | 0.142 | 3.596 | 0.10 | 0.099 | 0.221 | 0.035 | 0.060 | -8.426 | 0.695 | 0.699 | -0.348 |
| 1 v 2 | < 0.001 | 0.196 | 0.111 | 12.939 | 0.094 | 0.076 | 6.858 | 0.079 | 0.077 | 0.550 | 0.632 | 0.735 | -12.282 |
| 1 v 4fold | < 0.001 | 0.196 | 0.170 | 3.138 | 0.094 | 0.10 | -1.745 | 0.079 | 0.035 | 14.416 | 0.632 | 0.695 | -6.860 |
| 2 v 4fold | < 0.001 | 0.111 | 0.170 | -8.665 | 0.076 | 0.10 | -8.079 | 0.077 | 0.035 | 12.882 | 0.735 | 0.695 | 4.716 |

**Evidence for selection against nonsense mutations**

Why might selection act differently on different mutations at different sites? We have observed from analysis of 4-fold sites a strong C|G->U mutation bias in SARS-CoV-2 (Rice, et al. 2020) (Fig 1). The above evidence suggests that at first sites within codons there is especially strong contemporaneous selection to counter this mutation bias. Why might this be? In all genomes premature stop codons generated by nonsense mutations are commonly under strong purifying selection and there is no reason why this shouldn't apply to SARS-CoV-2. Indeed, intra-host mutation appears to generate nonsense mutations that fail to transmit (Tonkin-Hill, et al. 2020).

N->U mutations at codons NAA, NGA and NAG will generate stop codons (where N can be A, C or G). The nine codons should be at a frequency of 9/61 = 14.75% under unbiased nucleotide content but are at 17.05% with AAA (3.76%) being the second most common codon after GUU (3.9%). Mutations to U at the second site can never generate a stop codon. Consistent with these expectations, the reduction in U seen at non-4-fold sites compared to 4-fold sites is profound at site 1 (Z=-6.86) but not seen at site 2 (Z=4.72) (Table 1). Similarly, site 1 has much less predicted U content at equilibrium than site 2 (Z=-12.3). The raw predicted U content at equilibrium reflects these trends: U1*=63.2%, U2*=73.5%, U3*=70.1%, U4* = 69.5%. More specifically, when considering the full mutational profile of the virus, we find nonsense mutations to be significantly less common than other point mutations (2x2 $Chi^2$; $\chi$= 1942.9, df = 1, $p < 2.2 \times 10^{-16}$). They are also less common when they generate an in-frame stop codon than a +1-frameshifted (2x2 $Chi^2$; $\chi$= 1924.4, df = 1, $p < 2.2 \times 10^{-16}$) or a +2-frameshifted (2x2 $Chi^2$; $\chi$= 2626.1, df = 1, $p < 2.2 \times 10^{-16}$) stop, and are significantly more likely at the first nucleotide position than the second (2x2 $Chi^2$; $\chi$= 137.1, df = 1, $p < 2.2 \times 10^{-16}$). The commonality of nonsense mutation at first sites is likely owing to the strong N->U mutation bias, all stop codons having U at the first site.

Sequenced isolates deposited in GISAID are usually consensus sequences that discard all but the most frequent base at any position from individual samples, and therefore likely do not fully reflect the diversity of SARS-CoV-2 among infected individuals. To gain insight into within-individual variation, we analysed variants identified from publicly available SARS-CoV-2 raw sequencing read data to quantify variants within samples. Could this data provide evidence for missing mutations in GISAID sequences and purifying selection being a reason? Counting nonsense mutations present at some frequency in 1,092 samples, there is a mean of 0.23 nonsense mutations per sample. Compared to GISAID isolates, within-individual samples are far more likely to harbour a nonsense mutation (1.3% of GISAID isolates vs. 13.4% of within-individual samples, 2x2 $Chi^2$; $\chi = 1110.3$, df = 1, $p < 2.2 \times 10^{-16}$). Similar to the mutational profile above, for within-individual variation, first nucleotide positions are significantly more likely to generate an in-frame stop codon than second positions (0.9% vs 0.6%, respectively, 2x2 $Chi^2$; $\chi=7.0$, df = 1, p = 0.008).

**Nonsense mutations account for ~10% of missing mutations**

A prior observation of Ka/Ks ~1 (Bai, et al. 2020) suggests that nearly all intra-host selection must be against nonsense mutations. Selection against nonsense mutations cannot, however, explain all the observed patterns. Under the assumption that there is no selection to avoid out-of-frame stop codons, we may extrapolate the out-of-frame nonsense mutation rate to estimate how many nonsense mutations are missing in the above trends. Taking the out-of-frame per-trinucleotide nonsense mutation rate as the mean of the +1 and +2 frameshifted rates, this equals $1.46 \times 10^{-5}$ mutations per trinucleotide compared to $1.26 \times 10^{-6}$ in-frame. We are hence missing nonsense mutations at a rate of $1.46 \times 10^{-5} - 1.26 \times 10^{-6} = 1.33 \times 10^{-5}$ per trinucleotide and, scaled to the number of in-frame trinucleotides analysed that are one point mutation away from a stop, this equates to 3,205 missing nonsense mutations. As we above estimate a total of 31,797 mutations missing from the sequence data, nonsense mutations only account for approximately 10.1% of these.

We also used an alternate method of estimating the expected number of missing nonsense mutations analytically, relying on trinucleotide substitution patterns observed at 4-fold degenerate sites. As we have previously mentioned, 4-fold degenerate sites should evolve in a mostly neutral way and as such, the observed mutation rates on these sites should better reflect mutational bias. For this, we compared the proportion of in-frame nonsense mutations observed in our dataset

(264 nonsense mutations out of 49,358 trinucleotide changes), to an expected proportion of nonsense mutations derived from distributing this same number of mutations randomly across the sequence at the rate of trinucleotide substitutions of 4-fold degenerate sites (an average of 2909.362 nonsense mutations out of 49,358 trinucleotide changes, 95% CI lower = 2908.410, upper = 2910.314). This comparison equates to approximately 2645 missing nonsense mutations on average, accounting for only 8.3% of our 31,797 estimated missing mutations. This is close to the above estimate of ~10%. Given prior evidence that Ka/Ks =1 (Bai, et al. 2020), this result is surprising, suggesting that the majority of counter selected mutations are not nonsense ones.

Reinforcing this result, we also see that when all 12 mutational types are considered, not only do 4-fold degenerate sites have the highest rate in 22/24 comparisons (binomial test, $P=3.6 \times 10^{-5}$) but the rate is also higher at 4-fold degenerate sites for mutations that could never generate stop codons e.g. G->C, U->C at sites 1 and 2 (Fig 1). Likewise, G->U rates are marginally higher at site 1 rather than site 2, whereas we expect the opposite if all selection is against nonsense mutations.

While second site nucleotide content is considered the key determiner of the chemical property of the encoded amino acid (Haig and Hurst 1991; Freeland and Hurst 1998; Gilis, et al. 2001; Schwersensky, et al. 2020), only five of twelve first site vs second site comparisons have higher rates at the first site. The same analysis of the 12 mutational types emphasises the great disparity in G->U, and to a lesser degree C->U, mutation between 4-fold degenerate sites and codon sites 1 and 2, this despite the fact that some (Leu->Leu) first site C->U mutations are synonymous (Fig 1).

What then might predict these trends? We start by considering parameters that might explain why some amino acid exchanges are seen less than expected given the mutational profile. Then we consider in more detail a biophysical model of disruption of a key protein-protein interaction, spike with ACE2.

**Amino acid cost and chemical distance as predictors**

Are there general properties of the missense/nonsynonymous mutations that are under-represented compared with a mutational null? In order to test this, we first analysed the relationship between under/over-representation of amino acid substitutions and 12 estimators of different biochemical properties of such amino acids (Supplementary Table 2). However, as mentioned, mutational biases can occur in the context of more than one nucleotide, for example when responding to codon bias or as a result of non-selective mutational processes, like APOBEC induced genomic C-to-U deamination (Simmonds 2020b). To account for the effect of multi-nucleotide mutational biases on amino acid replacements, we first measured the over/under-representation of each pair of amino acid replacements, compared to expectations derived from trinucleotide substitution patterns observed at four-fold degenerate sites. Then we used a Best subset regression to select an optimal linear model explaining the over/under-representation of amino acid substitutions using the 12 estimators of biochemical properties, plus a set of variables measuring the degree in change in U nucleotide, as well as UU and CG dinucleotide content between codons in each pair of amino acids.

The optimal model found includes many parameters indicative of selection against non-synonymous mutations that break proteins by replacing one amino acid with a chemically dissimilar one. Notably, between pairs of amino acids, predictors include their distance in a BLOSUM100 similarity matrix, differences in polarizability and residue volume (Adjusted $R^2$ = 0.3533, p-value = $9.563 \times 10^{-11}$, Supplementary table 3). Perhaps more enigmatically, we also observed an enrichment of missense mutations to amino acids with a slower decay, possibly suggesting some selection for reduced metabolic cost of SARS-CoV-2 protein production (NB fast decay means more cost per unit viable amino acid). There is avoidance of UU residues but this isn't significant.

**Spike-ACE2 interaction disruption predicts missing mutations**

The above measures are fairly broad brush but suggest, as might be expected, protein disruption to be a source of purifying selection in real time. Using spike protein, for which we also have an underlying biophysical model of its binding (Starr, et al. 2020), we can examine the same hypothesis with better granularity. For this we again compared within-individual variation to GISAID isolates. Firstly, counting observed missense mutations in the receptor binding domain of the spike (S) gene, we find 212 unique amino acid substitutions in our GISAID alignment compared to the reference sequence and 61 substitutions in the within individual variation. This is especially notable as the number of GISAID isolates in our alignment (83,665 non-reference isolates) with the reference sequence is many times the number of samples with observed variants in the Galaxy Project within-individual variation dataset (1,092 samples). Secondly, using a mutational screen of amino acid substitutions in the receptor binding domain and their measures of relative ACE2 binding activity compared to the reference genome (Starr, et al. 2020), we compared the phenotypic effects of the substitutions we observe in GISAID isolates and those from within-individual variation (Figure 3). Substitutions observed within individuals reduce relative ACE2 binding activity more than observed GISAID substitutions (Median binding activity respectively: -0.27 and -0.08; p = 0.0002; Wilcoxon ranked-sum test). This provides evidence for unobserved SARS-CoV-2 variation when considering sequenced GISAID isolates only and purifying selection being a possible reason for such variants failing to reach the most frequent nucleotide at a given position and therefore discarded at the consensus sequence stage.

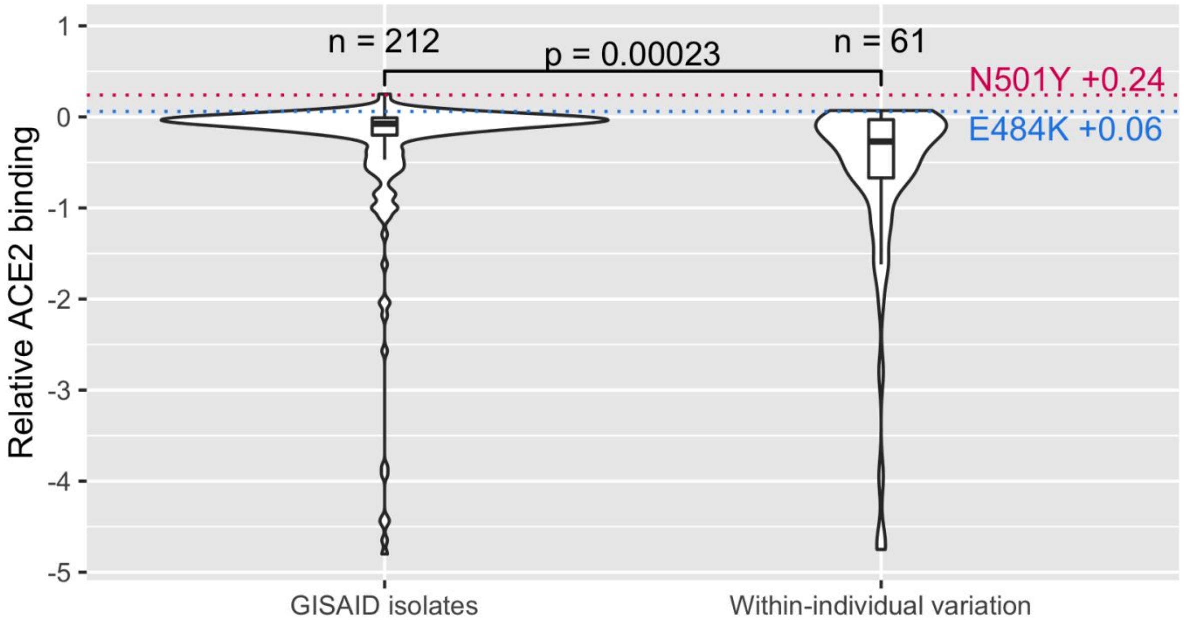

**Figure 3. Relative effects on ACE2 binding activity for missense mutations in GISAID isolates and within individual variation.** Distribution of relative effects on binding activity of unique missense mutations within the receptor binding domain that are observed one or more times in GISAID isolates and Galaxy Project within individual variation. Change in relative ACE2 binding of two notable amino acid substitutions within the receptor binding domain of spike observed in variants of concern, N501Y and E484K, are annotated as dotted lines.

**Synonymous mutations degrading match to the human codon usage are counter selected**

Above we have concentrated on what a priori are expected to be relatively large effect mutations. We can also ask whether we can also detect selection at synonymous sites. In order to test this we compare the proportion of 4-fold synonymous mutations resulting in a codon with an increase, decrease or with no effect on optimal codon usage. At first sight, one might imagine that such a method could not work as we are attempting to infer selection at synonymous sites using observed mutations at synonymous sites, rendering the analysis circular. However, this need not to be true. Consider two amino acids for which the "optimal" codon for each has a different synonymous site. If one amino acid has a U as the optimal synonymous site then common C->U mutations will not be opposed by selection. However, if another amino acid has C as the optimal site then the same mutation will be opposed by selection. If selection is strong enough then both processes will contribute to the net observed mutational matrix. Consequently, while for the two the different synonymous sites with the same nucleotide content the null rate will be the same, we expect to see deviations away from this null in a manner dependent on whether mutation bias and selection are aligned or not. Deviations between expected mutational profiles and observed mutational biases, then have the potential to detect selection on synonymous mutations. The method is flexible to any definition of "optimal" as we can test whether deviation from the observed mutational null tends to act against mutations that are thus defined as non-optimal. We consider two such definitions.

First we consider translational efficiency, as measured by the tRNA adaptation index (tAI)(dos Reis, et al. 2004) in humans, calculated based on the copy number of tRNA genes and the binding strength between a codon and a tRNA (Yoon, et al. 2018) with random expectations derived from simulations taking into account the trinucleotide mutational patterns of 4-fold sites. Using tAI as a measure for selection on translational efficiency has some pitfalls. In multicellular organisms with larger genomes, there is no correlation between codon usage and tRNA, possibly due to a higher tRNA gene redundancy in larger genomes, which would decrease selection for specific codons (dos Reis, et al. 2004). Furthermore, tRNA copy numbers do not necessarily reflect the fact that pools of distinct tRNAs are dynamic and can vary considerably in different conditions and tissues (Hernandez-Alias, et al. 2021). We observe a significant depletion of 4-fold synonymous mutations increasing codon adaptation (two tailed p-value = 0.0022, Supplementary Figure 1), as well as a small, yet not significant, enrichment of mutations that decrease or don't disrupt tAI (two tailed p-value = 0.0984 and 0.326, respectively). These results suggest, if anything, some selection acting against translational efficiency dependent on the tRNA pool.

Second, we compared the number of mutations that caused a switch in the SARS-CoV-2 genome to a codon with a higher relative synonymous codon usage (RSCU) in human. When accounting for the trinucleotide mutational patterns, which would better capture the effects of mutational biases derived from CpG avoidance or APOBEC induced mutation cytosine deamination, we do observe a significant over-representation of mutations that increase RSCU (p-value = $1x10^{-4}$, Supplementary figure 2). This result is consistent with selection occurring in SARS-CoV-2 to match the human codon usage profile.

We further ask whether such signatures of selection can be detected within hosts. For this, in a similar manner as with the between host data, we compared the observed tAI and RSCU of fourfold synonymous positions in the intra-host dataset, against random expectations derived from simulations taking into account the trinucleotide mutational patterns of 4-fold sites in this same data. We find a significant depletion of 4-fold increasing codon adaptation (two tailed p-value = 0.0044, Supplementary Figure 3), as well as a significant enrichment of mutations that don't disrupt tAI (p = 0.0354). This is consistent with the above finding of selection against tRNA dependent translational efficiency. We also detect over-representation of synonymous mutations increasing human RSCU in the SARS-CoV-2 intra-host data but the deviation from null is not significant (Supplementary figure 4). While there should be some selection occurring among strains within a host, reflected in differences in allelic frequency, selection on RSCU might not be strong enough to have a measurable impact at the shorter time scale reflected by intra-host variation.

## Discussion

Prior to the genomic age mutation rates were classically estimated by considering substitution rates (between two species) at synonymous sites with assumptions made about generation times and time to common ancestry to provide a per generation per base pair estimate (see e.g. Keightley and Eyre-Walker 2000). The restriction to the synonymous sites was a means to reduce the impact of purifying selection depressing the estimate. More recently, this method has been supplanted by MA line or parent-offspring sequencing (Lynch, et al. 2016). Such methods assume that there is no important degree of purifying selection between parent and recent descendants and hence that the profile and rate of mutations can be estimated in an unbiased manner. Our finding of common and strong purifying selection detectable in real time affecting mutations prior to their being sequenced strongly suggests that, at least for SARS-CoV-2, this is not the case. In principle our null simulation correction method could also be employed to correct for underestimation in MA and parent-offspring analyses to determine the mutation rate. However, in genomes such as that of humans, where few sites are subject to purifying selection, the correction is probably not important. For more economical genomes (with higher CDS density) it may be more relevant.

Given the evident purifying selection, an estimate of the rate of evolution of the virus is not the mutation rate *sensu strictu*, but rather of the rate at which new mutations appear and are viable enough to be sequenced. The latter measure is sometimes referred to as the substitution rate (van Dorp, et al. 2020), the rate of evolution (Hill and Rambaut 2020) or the mutation rate (Zhao, et al. 2004; Pathan, et al. 2020). Given our results we advise against the latter usage to avoid confusion. Put differently, if one were to take estimates of rates of sequence change for SARS-CoV-2 that employ observed RNA changes (Duchene, et al. 2020; Hill and Rambaut 2020; Nextstrain 2020), and assume that this is the underlying mutation rate, one would be wrong. We indeed find that the discrepancy is not modest (an ~50% correction would be needed).

While to estimate the true underlying mutation rate we thus need to control for purifying selection, the discrepancy between the mutation rate (*sensu strictu*) and the evolutionary rate is important in other contexts. If selection on viral escape from vaccines (or antiviral drugs) is in part owing to intra-host selection, then knowing the underlying mutation rate, and the difference between it and the apparent evolutionary rate, is important. Furthermore, claims of higher or lower mutation rates in some lineages would need to control for the possibility of differences in, for example, effective population size (Ne) or sampling depth. Variation in Ne, modulating the strength of selection, could result in conflation of differences in the mutation rate *sensu strictu* with efficacy of selection differences (lower Ne permits more mutations to circulate). Similarly, we would expect that deeper sampling of genomes within an individual will provide evidence for genomes that will be removed by purifying selection but have yet to be removed (as indeed we show). This could also lead to misleading inference of increased mutation rates. To understand how important within-host selection might be, it is important to control for such effects and unbiased sampling of 4-fold degenerate sites is, we suggest, preferable to analysis of sequence classes known to be under purifying selection.

The analysis of missing mutations is, however, of less interest in contexts where we wish to employ the rate of evolution to estimate coalescent times, as in this context the appearance rate (circulating in the population) per unit time is the relevant metric, not the true underlying mutation rate. Nonetheless, in this context understanding whether there are sites subject to purifying selection can be important for determining whether rate estimate correction is needed. As O'Fallon (2010) noted, purifying selection acting at many linked sites can systematically bias genealogical reconstruction but by allowing a class of sites to have a time-dependent rate can enable some degree of correction. Likewise, Wertheim et al. (2011) show that, for other viruses, adjusting codon models to allow for purifying selection can lead to estimates of the time to common ancestry longer than those supposed from rates of observed circulating mutations. Our results suggest that such adjustments are then required for SARS-CoV-2.

Our new estimate is likely to be an under-estimate. Although we have attempted to control for nucleotide biases and biases in rates of each class of mutation, we have also assumed that 4-fold sites are themselves free from selection. Our analysis of two specific models of selection on codon usage provided no evidence for selection on codon usage to match tRNA pools (indeed selection appears to be in the opposite direction) but of selection to match human codon usage. The later result was seen unambiguously when testing the circulating genomes for deviation from null, but not statistically significantly replicated with intra-host variation data.  However, SARS-CoV-2 has multiple modes of selection on nucleotide content that would not be detected by such methods. These include selection against CpG dinucleotides to avoid ZAP, against UpA to avoid RNAase L and more generally against U, mediated possibly by transcript destabilization and/or expression level (Rice, et al. 2020). Just as we observe possible selection against U so we and others have identified possible selection for A (Kustin and Stern 2020; Rice, et al. 2020). One possible mechanism of this could indeed reflect the high U content and hence selection for A to enable stable base pairing in RNA stem structures (Ratcliff and Simmonds 2021).

Some of our results on the causes of purifying selection seem fairly simple to interpret. It is not surprising that nonsense mutations are counter-selected, nor that a biophysical model of spike protein function recovers a trace of purifying selection. Similarly, that features like chemical similarity predict amino acid exchange rates make sense, as highly different amino acids are likely to corrupt proteins just as nonsense mutations do. Nonetheless, our results hold a few surprises when considered against the prior literature. While purifying selection was previously identified (see e.g. Tonkin-Hill, et al. 2020; Lythgoe, et al. 2021), given prior Ka/Ks estimates near unity (Bai, et al. 2020), seen also for SARS-CoV (Zhao, et al. 2004), it might reasonably have been inferred that most of the missing mutations must be nonsense mutations. Our results don't support this. We however consider Ka/Ks an unsuitable tool for analysis of polymorphic data, especially in a context with complex mutation and nucleotide biases (see introduction).

It is similarly, not so obviously expected that amino acid cost determinants (amino acid decay rate) would factor as predictors of amino acid exchange rates, with selection against more costly ones. The usual logic is that making "costly" amino acids, when cheaper good alternatives are available, causes a fitness cost owing to differential ATP usage. For amino acids with high decay rates, these costs are suffered more as the pool of amino acids needs replenishing faster. However, why a temporary visitor to a cell (the virus) that causes damage regardless, will have selection to use less costly amino acids is not so transparent. Why would it be under selection to use less costly amino acids if the cell making those amino acids will soon be dead anyway? In what sense would the virus benefit from using cheaper amino acids? The key amino acid parameter, decay rate rather than synthesis cost per se, may point to an alternative cause. There could well be selection for rapid viral replication. A genome that both harms the cell's ability to manufacture new amino acids but needs rapid translation, may be under selection to use those amino acids that have a long half-life, regardless of ATP costs. Usage of those with a short half could leave the virus slowed in translation waiting for ever rarer and diminishing pools of charged tRNAs. We thus suggest that amino acid ATP cost per se is not the key parameter, but rather delay to translation might be. That SARS-CoV-2 interferes with the host's splicing and translational machinery (Banerjee, et al. 2020), suggests that amino acid biosynthesis may well be affected.

Similar logic may explain why selection on synonymous sites failed to identify adaptation to the tRNA pool. Our estimation of this pool from tRNA copy numbers may well not reflect the pool of charged tRNAs as certain amino acids, with high decay rates, are limiting. Exactly why matching the human codon usage does matter is less clear, but a direct coupling between GC content and gene expression in both nuclear and cytoplasmic compartments (for reasons unknown) of virus-mimicking intronless transgenes (Mordstein, et al. 2020; Mordstein, et al. 2021) could underpin such an effect.

We highlighted several analytic challenges associated with this virus's genome. One we have not fully broached is the problem of potential interactions between genomic location, RNA structure and both mutation rate and mutation profile. We have controlled for complex mutational biases by consideration of di and trinucleotide context. We have also attempted to control for rate heterogeneity by exclusion of hyper-mutagenic sites, much as previously we excluded homoplasic sites (Rice, et al. 2020). Hyper-mutagenic sites are relatively rare (1% of all sites, 1.8% of variable sites, 2.7% of 4 fold sites, 4.2% of variable 4-fold sites) but given that they contribute a disproportionate number of observed mutations they have the potential to lead to false inference if the mutational spectrum at such sites is different from that at non-hyper-mutagenic sites. While the sample of hypermutagenic sites is limited, we can compare their trinucleotide context with that of the remaining mutations for fold-fold sites (Supplementary Figure 5). We find relative enrichment of UCN->UUN consistent with more frequent activity of APOBEC on hypermutable sites. We also see evidence for enrichment of CGN->CUN. This is suggestive of selection against CG residues, possibly owing to ZAP mediated attack. However such a model would also predict CGN-> C[C|A|U]N which we don't see. A possible combination of mutation bias (towards U) and selection against CG might need to be evoked.

Our method to control for hyper-mutagenic sites defined sites by reference to the number of independent mutational events seen across all sites, with hypermutagenic being defined by deviation from a negative binomial. This method, however, makes no allowance for position by nucleotide effects. One could suggest that there might be sites that don't have unusually large numbers of mutations compared to all other sites, but do when considering their ancestral nucleotide state. We have considered such a model treating each of the four nucleotides independently and eliminating, for each, those sites in the alignment with more independent mutational events than expected given a negative binomial distribution parameterised for the nucleotide in question. To assess whether this alternative methodology makes a difference to the final analysis of the residual mutational matrix (i.e. after removal of hyper-mutagenic sites) we compare the residual matrix from the nucleotide controlled and uncontrolled methods. We find no significant difference between the two residual matrices (P = 0.897: Predicted equilibria for original hypermutable threshold - A: 0.170, C: 0.100, G: 0.035, U: 0.696; Predicted equilibria for nucleotide-controlled thresholds - A: 0.162, C: 0.076, G: 0.025, U: 0.738).

Adding to such complexity is the notion that the rate or profile of any given nucleotide motif may be contingent on its genomic location e.g. in a stem loop or not. Untangling cause and effect in this instance will not be trivial. A low rate of observed SNPs in RNA stem structures (Simmonds 2020a) could, for example, reflect selection against mutations that disrupt RNA stem structures (Simmonds 2020a). Alternatively, it may be owing to a reduced mutation rate if RNA stems protect from mutation, for example via shielding from APOBEC (Ratcliff and Simmonds 2021). We are unaware of theoretical work that attempts to correct for motif (*k* mer) by location effects on rates and profiles. This we leave to future work.

## Materials and Methods

### Creating a mutational matrix

Multiple sequence alignment of 106,448 SARS-CoV-2 genome assemblies was downloaded from the GISAID (Shu and McCauley 2017) Initiative EpiCoV platform, these being those available as of 28 September 2020. Isolates with more than 1% of ambiguous base calls or more than 5% of any CDS missing were removed. This left 83,666 genomes. For list of genomes and sources see Supplementary Table 1 and Supplementary data 1.

We employed NCBI Reference Sequence NC_045512.2 to specify CDS coordinates. However, following further annotation of genes (Kim, et al. 2020), we modified the gene locations to reflect those specified: https://github.com/hyeshik/sars-cov-2-transcriptome/blob/master/reference/SARS-CoV-2-annotations.gff. Specifically, to avoid a small codon overlap, we exclude CDS overlaps, hence employed annotation:

ORF7a protein 27394.27759→27394.27753

ORF7b protein 27756.27887→27762.27887

To consider ORF1a and ORF1b independently and to avoid overlap, we employ:

ORF1a→266-13465

ORF1b→13471-21552

CDSs for each gene in each strain, were extracted from these alignments, and frameshift correction was then applied using the protein sequence of the Wuhan-Hu-1 reference genome (EPI_ISL_402124), sampled from a retailer at Huanan Seafood Wholesale Market, Wuhan on December 30, 2019 as reference, using the DECIPHER R package. This early sequence matches the consensus generated from all of the 19 sequences that were collected prior to December 31. CDSs were then translated, realigned with MAFFT 7.458 (Katoh and Standley 2013), and then reversed translated using TranslatorX (Abascal, et al. 2010).

A phylogenetic tree of SARS-CoV-2 isolates (released 28th Oct 2020, (Lanfear 2020)) was pruned using DendroPy v4.4.0 (Sukumaran and Holder 2010) to match isolates present in our sequence alignment, and similarly our sequence alignment was filtered to match isolates present in the phylogenetic tree. This left 78,971 genomes present in both. Aligned CDSs were concatenated to create a single coding sequence alignment of length 30,696 bp as input for ancestral sequence reconstruction. Ancestral sequence reconstruction at internal nodes of the predefined phylogenetic tree was performed using an empirical Bayesian method with a GTR+G model of substitution in IQTree v2.1.2 (Minh, et al. 2020). Inferred bases with a probability of less than 0.99 were masked.

Known problematic sites in the SARS-CoV-2 genome (released 12th December 2020, available from https://github.com/W-L/ProblematicSites_SARS-CoV2/blob/master/problematic_sites_sarsCov2.vcf) identified and collated at https://virological.org/t/masking-strategies-for-sars-cov-2-alignments/480 were masked and the number of mutations per site at four-fold degenerate sites were counted.

Given that some sites appear to be both hypermutable, hence subject to homoplasy (van Dorp, et al. 2020), and potentially unrepresentative of the rest of the genome we sought to exclude these sites from more general analysis (we consider their properties separately). To find thresholds for masking hypermutable sites in the genome, a negative binomial distribution, with mu fixed to the median number of mutations per site (median: 1), was fitted to the observed values using the fitdist function of the fitdistrplus R package (fitted distribution: mu = 1, size estimate = 0.3414126 (Delignette-Muller and Dutang 2015)). An expected number of hypermutable sites can be estimated from the fitted distribution for a given number of sites. We set a cut-off threshold where we expect no more than one site with that number of mutations and mask the sites above that threshold. For example, for 4,248 four-fold degenerate sites, we expect at least one site with 17 mutations and less than one site with 18 mutations, and therefore mask sites where 18 or more mutations have occurred independently across the tree. For 9,739 first, second, or third codon position sites, we expect at least one site with 19 mutations per site, etc.

We also consider a second approach in which we define (and exclude) hypermutagenic sites by reference to the number of mutated sites with the same pre-mutation nucleotide. That is to say, for each site, we determine the number of independent mutational events at that site. We then compare these by-site numbers to other sites within the alignment with the same pre-mutation nucleotide. We then calculate the mean number of independent mutational events for all such sites of a given pre-mutation nucleotide. The mean of this distribution then informs an expectation based on a negative binomial. We again set a cut-off threshold where we expect no more than one site with that number of mutations and mask the sites above that threshold. Under the first method 2.7% (116/4248) of four-fold sites are hypermutagenic and 4.2% (116/2798) of variable four-fold sites are hypermutagenic. Under this second, nucleotide dependent method 0.5% (19/4248) of four-fold sites are hypermutagenic and 0.7% (19/2798) of variable four-fold sites are hypermutagenic. 17 of these 19 hypermutable sites are considered hypermutable in the prior method too.

Mutations were counted from root to tips of the tree, taking ancestral parent nodes as reference and counting mutations in descendants at each node of the tree. If a mutation occurred at the same site in two descendants at the same position of the tree, this mutation was counted once (similar to De Maio, et al. 2021). When counting variants, known problematic sites within the genome were masked, hypermutable sites above their respective thresholds were masked, and codons containing more than one variant in a single genome compared to its direct ancestor were masked. Whole-genome nucleotide flux estimates were obtained by counting the frequency of each type of mutation and normalising by the frequency of the nucleotide in the reconstructed ancestral genomes. This resulted in a dataset of 51,244 variants.

**Estimating the number of "missing" mutations**

How many mutations would be expected if all codon sites evolved as if they are fourfold? To estimate this, and hence how many mutations might be missed in the sequencing data, let us suppose that the number of mutations at ancestral base N (N = A, C, G, or U) normalised to the number of ancestral Ns at 4-fold degenerate sites is $N_4$. Likewise, $N_1$, $N_2$, $N_3$ for codon sites 1 to 3 respectively. The absolute number of missing (*M*) mutations across the genome is hence:

$$M = \sum_{i=1}^{i=3} F(N^i).(N_4 - N_i)$$

where $F(N^i)$ is the absolute number of occurrences of nucleotide *N* as the ancestral residue at base *i* across all reconstructed sites in the tree.

The comparable sum of all mutations observed (*O*) is:

$$O = \sum_{i=1}^{i=3} F(N^i).\,N_i$$

Note here we use all mutations at third sites because we need to count all mutations. The true total (*T*) number of mutations then is: $T=O+M$. For every observed mutation the proportion missing ($P_m$) of those observed is: $P_m = M/O$.

We extend the same method to consideration of dinucleotide-defined mutation bias. There are, however, two complicating factors in such analysis: (i) dinucleotides may mutate at either of their nucleotide sites and (ii) any given point mutation will affect two overlapping dinucleotides (a mutation at B in ABC, is both associated with AB and BC). To address problem (ii) we calculate missing "dinucleotide changes", rather than mutations, the total number of which may be halved to estimate the number of missing mutations. To address problem (i) we control for each mutation's nucleotide position within the dinucleotide in our analysis.

For each of the 16 dinucleotides we first calculate six position-specific mutation rates: $D_{(1)2}$, $D_{1(2)}$, $D_{(2)3}$, $D_{2(3)}$, $D_{(3)1}$ and $D_{3(1)}$, where the numbers represent dinucleotide position within a codon and brackets indicate the mutation site. These we compare to the position-controlled fourfold null mutation rates. The number of missing dinucleotide changes (M) for dinucleotide 'D' may be hence be calculated at each position (12, 23 or 31) as:

$$M_{12} = \sum F(D^{12}).(D_{(4)1} - D_{(1)2}) + \sum F(D^{23}).(D_{2(4)} - D_{1(2)})$$
$$M_{23} = \sum F(D^{23}).(D_{(4)1} - D_{(2)3}) + \sum F(D^{23}).(D_{2(4)} - D_{2(3)})$$
$$M_{31} = \sum F(D^{31}).(D_{(4)1} - D_{(3)1}) + \sum F(D^{31}).(D_{2(4)} - D_{3(1)})$$

where $F(D^{12})$ is the number of occurrences of dinucleotide *D* as the ancestral residue at position 12. The total number of missing dinucleotide changes (M) is: $M = M_{12} + M_{23} + M_{31}$

The comparable sum of all dinucleotide changes observed, *O*, for dinucleotide 'D' can be calculated at each position (12, 23 or 31):

$$O_{12} = \sum F(D^{12}).(D_{(1)2}) + \sum (D^{12}).(D_{1(2)})$$
$$O_{23} = \sum F(D^{23}).(D_{(2)3}) + \sum (D^{23}).(D_{2(3)})$$
$$O_{31} = \sum F(D^{31}).(D_{(3)1}) + \sum (D^{31}).(D_{3(1)})$$

The total number of observed changes for dinucleotide D is calculated as: $O = O_{12} + O_{23} + O_{31}$
The true total for dinucleotide D is then: $T = O + M$

The true totals of each dinucleotide may be summed to estimate the true total number of dinucleotide changes. As point mutations affect two dinucleotides, we divide this value by two to predict the true number of mutations.

**Calculation of mutational equilibria**

Given that the mutational profile is strongly U biased, considering solely rates of GC<->AU mutations (Long, et al. 2018) is likely to miss important dimensions. The equilibrium content of all four nucleotides we therefore estimate using the full mutational spectrum (Charneski, et al. 2011; Rice, et al. 2020). We here follow the same methodology as used in our previous publication (see Rice, et al. 2020). Briefly, if the frequency of G is denoted *G* and the frequency of U is denoted *U*, etc. Mutational flux from G to U, per occurrence of G, is denoted *g2u*, and A to C, per occurrence of A is denoted *a2c*, and so on (each mutational flux captured by the mutational matrix). Equilibrium is then defined as occurring when the rate of loss of each nucleotide is equal to the rate of gain of the nucleotide, for all nucleotides, with the additional constraint that $A+U+C+G=1$:

$$G\,(g2u+g2c+g2a)=A\,(a2g)+U\,(u2g)+C\,(c2g)$$

$$C\,(c2u+c2g+c2a)=A\,(a2c)+U\,(u2c)+G\,(g2c)$$

$$A\,(a2u+a2c+a2g)=G\,(g2a)+U\,(u2a)+C\,(c2a)$$

$$U\,(u2g+u2c+u2a)=A\,(a2u)+G\,(g2u)+C\,(c2u).$$

**Comparing mutational matrices**

For each class of site (e.g. 4-fold degenerate, not 4-fold degenerate, codon first sites etc), we determine the absolute number of each of the 12 classes of mutation (A->C, A->U etc), the rate then being this normalised to the frequency of the ancestral base giving the rates (*n2m*) defined above, ie the rate of *n2m*, per incidence of *n*. We then analytically solve, using NumPy (Walt, et al. 2011), to determine the mutational equilibrium vector (of length 4), this specifying the frequencies of the four bases at mutation-neutral equilibrium.

To compare between pairs of equilibrium values (e.g. for codon $1^{st}$ sites and for 4 fold degenerate sites), we determine the Euclidean distance between the resulting vectors and perform randomizations. In these we randomly re-allocate the underlying mutations to pools the same size as contributed to the two vectors in the first instance. From each simulation we derive the equilibrium predicted values of the two pseudo mutational profiles and calculate the difference between them. From multiple simulations we determine the null distribution. We express the observed difference in terms of the distance away from the mean of the simulants in standard deviation units derived from the simulants (i.e. a Z score). The method permits both estimation of the significance of the distance between any two vectors and identification of the nucleotides most deviant (and the significance of each's deviation).

**Analysis of amino acid properties**

In order to test the relationship between over-representation of particular missense mutations and changes in the biochemical properties of amino acids we built a generalized linear model. We first started by calculating the bias in missense mutations as a Z-score:

$$ZAA_{bias.cod} = \frac{(O_{cod} - E_{cod})}{SD(E_{cod})}$$

$$ZAA_{bias} = \frac{\sum ZAA_{bias.cod}}{n_{cod}}$$

Where $ZAA_{bias.cod}$ is the mean measure of over/under-representation of change between codon pairs for each pair of amino acids: $O_{cod}$ is the observed number of single nucleotide substitutions switching from a particular codon to another for that pair of amino acids, and $E_{cod}$ is the expected number of codon changes when accounting for the rate of trinucleotide substitution at trinucleotides centred on 4-fold degenerate sites. $E_{cod}$ and its standard deviation were estimated as the mean of 10,000 simulations distributing 49,358 mutations randomly across the SARS-CoV-2 CDSs at the same rate as the trinucleotide substitution observed at 4-fold degenerate sites. The parameter $n_{cod}$ is the number of codon pairs resulting in a particular amino acid replacement. $ZAA_{bias.cod}$ values for each pair amino acids were then averaged to obtain a measure of over/under-representation of amino acid replacements, $ZAA_{bias}$. We then used a best subset regression, optimising for Bayesian information criterion, using the "bestglm" R package, to search for a subset of biochemical properties of amino acids (Supplementary table 2 for the full list of tested properties and references) that, on a generalised linear model, would best predict $ZAA_{bias}$.

**Estimate of expected nonsense mutations**

We used the same method as above, in order to calculate an estimate of the expected proportion of nonsense mutations. Briefly, in order to obtain the expected number of codon changes into a stop codon, when accounting for the rate of trinucleotide substitution at trinucleotides centered on 4-fold degenerate sites, we performed 10,000 simulations distributing 49,358 mutations randomly across the SARS-CoV-2 CDSs at the trinucleotide substitution rates centered around 4-fold degenerate sites. We additionally employ a method using the rate of out of frame mutations to UAG, UGA or UAG.

**Analysis of tRNA adaptation and codon usage bias**

To test if there is any evidence of selection on translational efficiency at 4-fold synonymous sites, we measured the difference in human tRNA adaptation index (tAI) and codon usage bias caused by each of the 4-fold degenerate synonymous mutations identified in our analysis (4,064 variants) when compared against the SARS-CoV-2 reference genome. tRNA adaptation index per codon were obtained from the STADIUM database (Yoon, et al. 2018) and codon usage tables were obtained from the CoCoPUTs database (Alexaki, et al. 2019). In order to measure if any particular type of change is overrepresented when compared to random expectations, we generated 10,000 simulations of 4,064 variants across all 4-fold degenerate synonymous codons in the SARS-CoV-2 reference genome, at the same rate as the nucleotide substitution observed at 4-fold degenerate sites. P-values of over-representation of each type of mutation were calculated numerically from comparing to the distribution of these simulants.

In order to account for trinucleotide mutational biases, we repeated the simulation process accounting for the rate as the nucleotide substitution observed at 4-fold degenerate sites. We first masked any 4-fold degenerate synonymous variant that was followed by a mutation or an alignment gap in the first site of the next codon in a particular strain or if a hypermutable or problematic site occurred within the codon or the first site of the next codon.

Analysis of selection on translational efficiency on within-individual variation (data described below) was performed in the same way. Briefly, we measured the difference in human tAI and RSCU bias caused by each of the 4-fold degenerate synonymous mutations identified in the within-host dataset (1,208 variants), and compared it to random expectation derived from 10,000 simulations aleatorily distributing 1,208 variants across all 4-fold degenerate synonymous codons in the SARS-CoV-2 coding sequence, at the same rate as the nucleotide substitution observed at 4-fold degenerate sites in the within-individual variation dataset.

**Within-individual variation and receptor binding domain substitution analysis**

Within-individual variants generated by Galaxy and HyPhy developments Teams (Nekrutenko, et al. 2020) as part of the Galaxy Project SARS-CoV-2 data analyses (available at https://covid19.galaxyproject.org/genomics/4-variation/) were obtained from GitHub (https://github.com/galaxyproject/SARS-CoV-2/blob/4df1456e65367cf62c011c33d322643e79a9513e/genomics/4-Variation/variant_list.tsv.gz), updated on 29th May 2020 and accessed on 21st July 2020. Known problematic sites in SARS-CoV-2 sequencing were removed as in section 'Creating a mutational matrix' and only variants with allele frequency >5% were considered. Samples from the sequencing project with NCBI SRA Study Accession SRP253798 were removed prior to analysis as some samples from this study were noted as being dominated by C->U (>99% variants of some samples C->U, https://virological.org/t/gained-stops-in-data-from-the-peter-doherty-institute-for-infection-and-immunity/486). Nonsense mutations were already annotated as 'EFF[*].FUNCLASS = NONSENSE' and here were quantified per sample and at which position the mutations occurred in codons. To compare nonsense mutations at first and second nucleotide positions of codons, the number of codons that were one mutation from a stop codon were counted in the reference sequence (for first sites: NAA, NAG, NGA; for second sites: UNA, UNG) and a chi-square test was performed.

Effects on binding activity of single mutations within the receptor binding domain of SARS-CoV-2 spike protein were obtained from supplementary table 2 of Starr et al. (2020). The above alignment of GISAID SARS-CoV-2 isolates was used to quantify unique amino acid substitutions at positions within this region. Within-individual variants were filtered for those within the receptor binding domain and unique amino acid substitutions were quantified. This method has the advantage that the predicted mutational effect is called dependent on biophysics alone, rather than methods that employ sequence conservation and variant frequencies (e.g. Dunham, et al. 2021) that would render the present analysis circular.

**Acknowledgements**

This work was supported by the Wellcome Trust (fellowship 207507 to G.K.) and the European Research Council (advanced grant ERC-2014-ADG 669207 to L.D.H.). We acknowledge the providers of all the genomes (see Supplementary Table 1).

**Data availability**

All genomes used in the analysis are available from GISAID. Genomes employed are listed in Supplementary Data 1 and Supplementary Table 1.

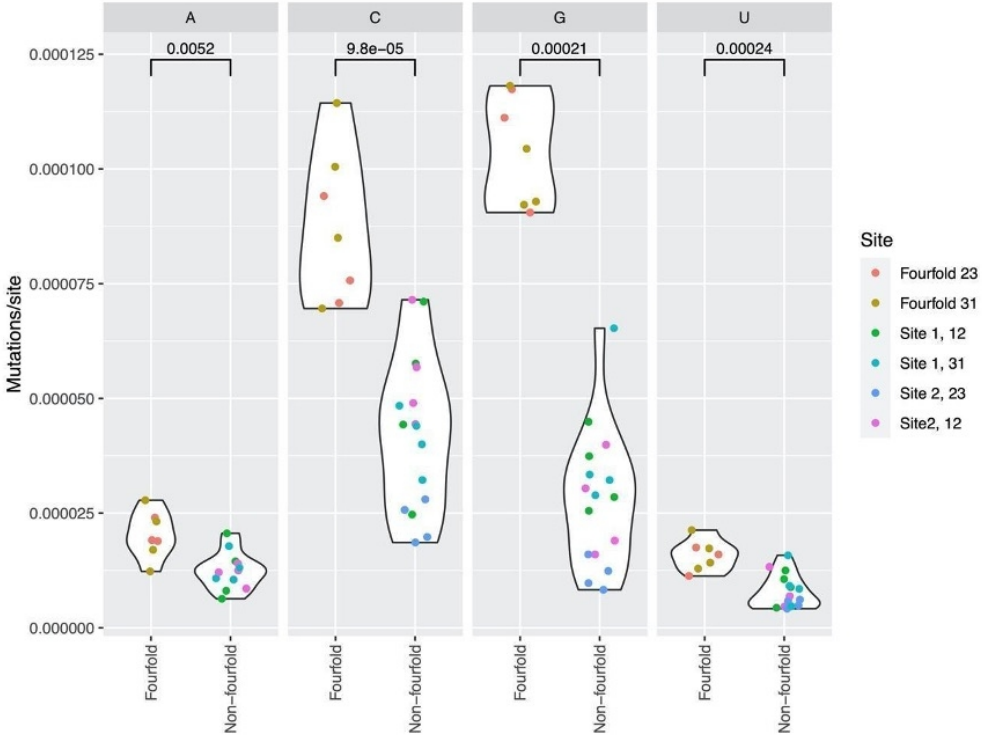

Figure 2

152x114mm (144 x 144 DPI)

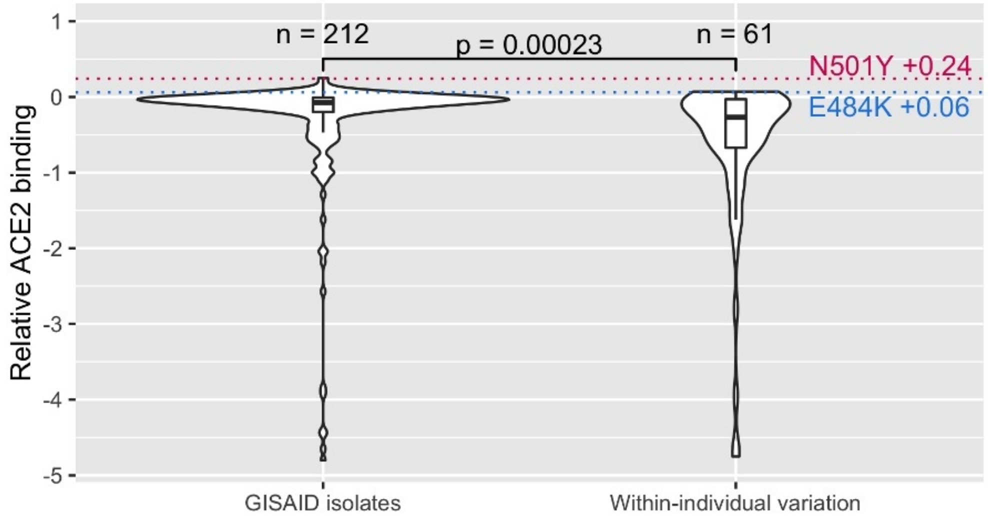

Figure 3

159x91mm (144 x 144 DPI)

## Supplementary Material

evab196_Supplementary_Data

