# Supplementary material for: Causes and consequences of purifying selection on SARS-CoV-2
Source: Genome Biol Evol. 2021 Aug 24:evab196. doi: 10.1093/gbe/evab196 (PMC8504154; doi:10.1093/gbe/evab196)
Supplement: evab196_Supplementary_Data [file evab196_Supplementary_Data.zip › suppl_data/Supplementary Figures copy.docx]

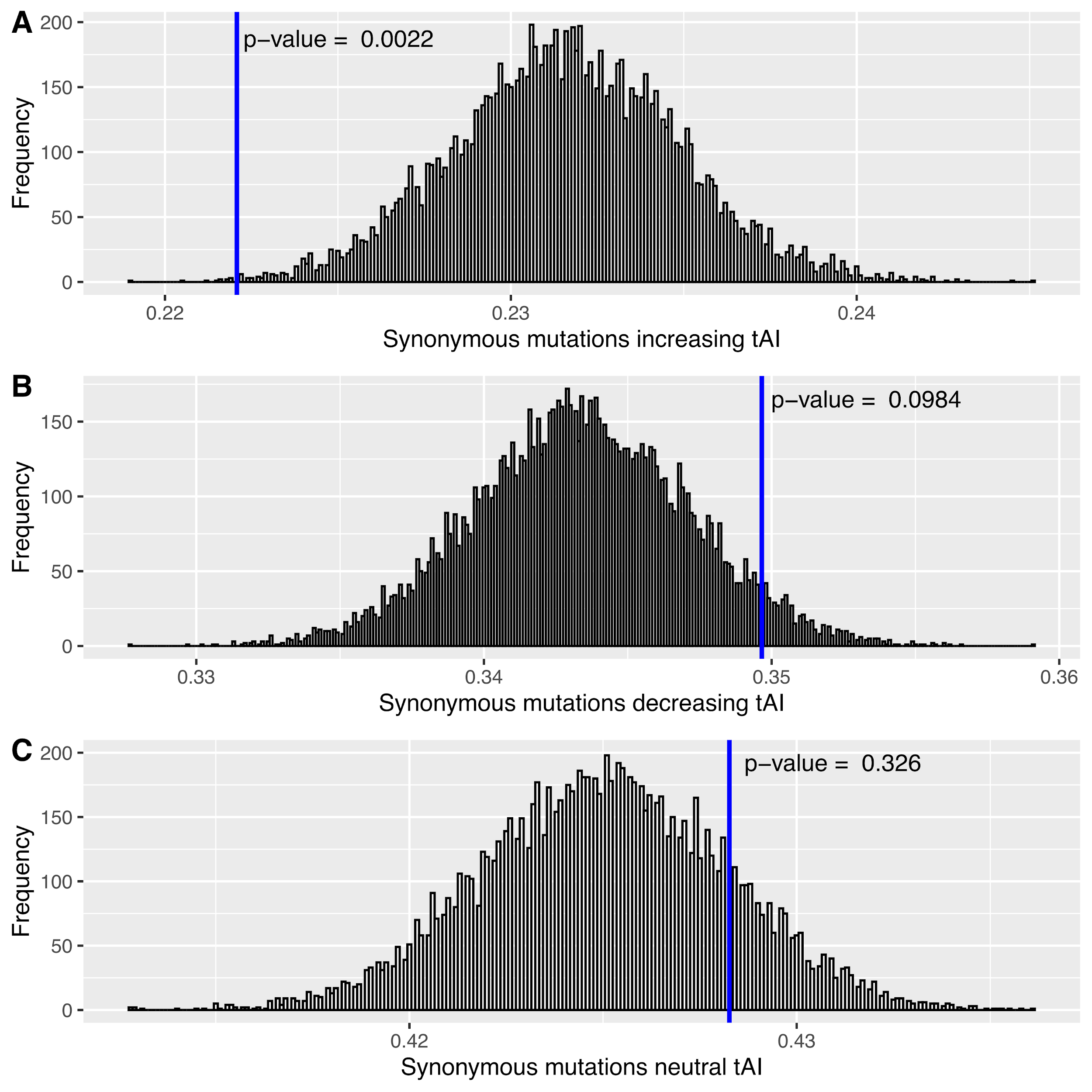


**Supplementary Figure 1. 4-fold Mutations in SARS-CoV-2 changing tRNA adaptation index when accounting for the trinucleotide mutational flux** Proportion of 4-fold synonymous substitutions in SARS-CoV-2 that increase (A), decrease (B) or don’t alter (C) the tAI of the codon (blue lines). Distributions represent the expected number of synonymous substitutions of each type obtained from random simulations taking into account the trinucleotide mutational flux.


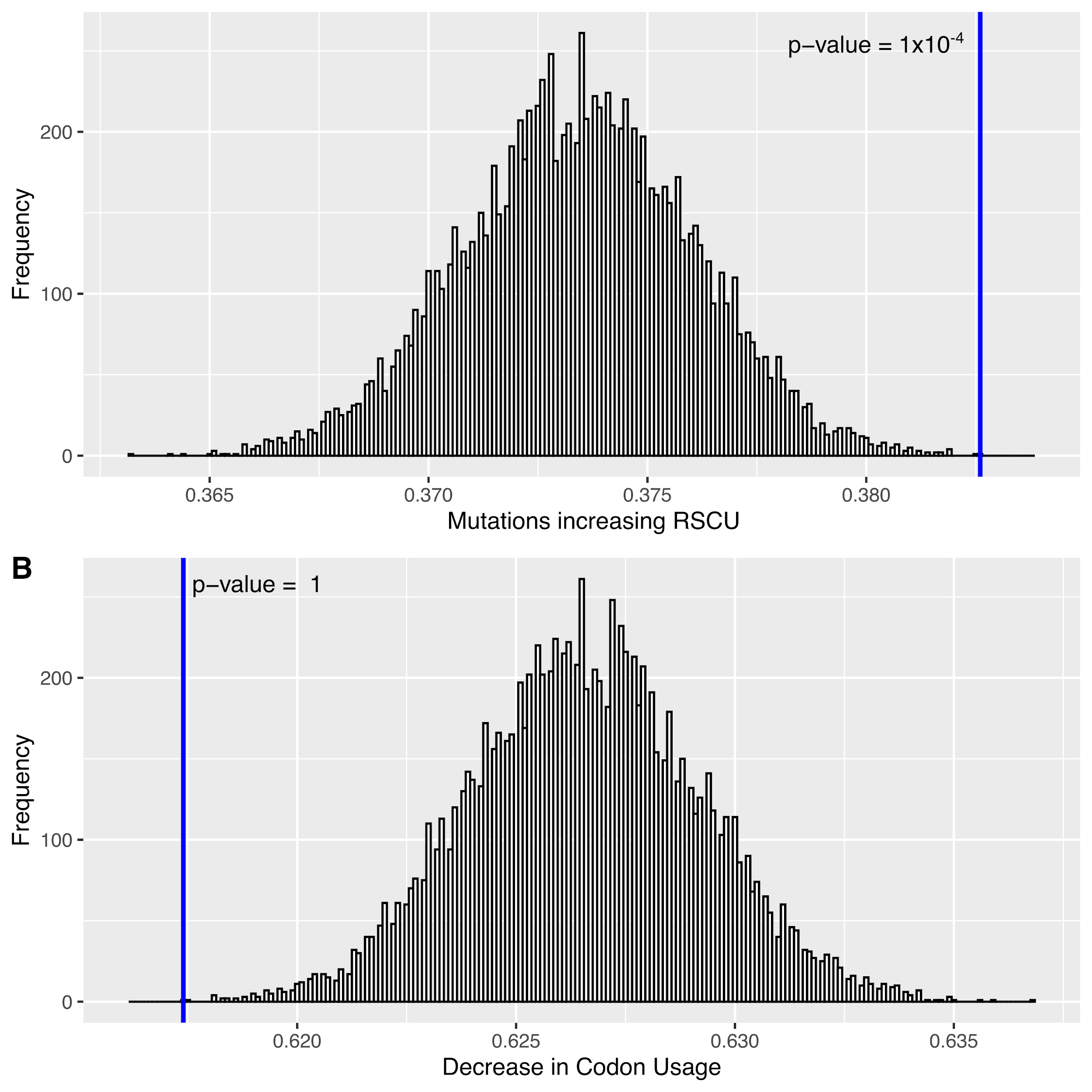


**Supplementary Figure 2. 4-fold Mutations in SARS-CoV-2 changing Relative Synonymous Codon Usage.** Number of 4-fold synonymous substitutions in SARS-CoV-2 that switch to a codon with a higher relative synonymous codon usage in human (blue line). Distribution of expected number of synonymous substitutions that increase RSCU obtained from random simulations taking into account the trinucleotide mutational flux.

**
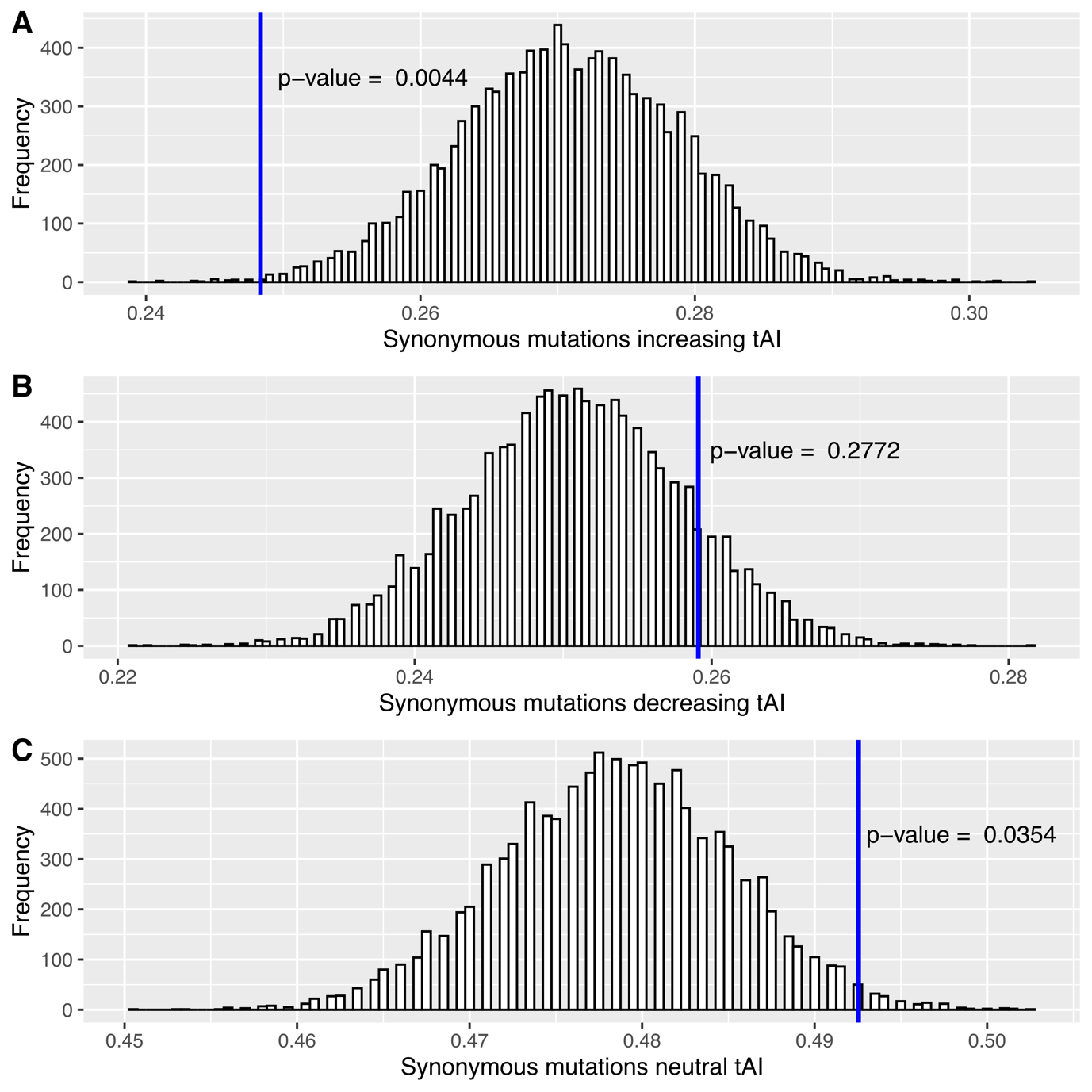
**

**Supplementary Figure 3. 4-fold Mutations in within host SARS-CoV-2 changing tRNA adaptation index when accounting for the trinucleotide mutational flux** Proportion of 4-fold synonymous substitutions in the SARS-CoV-2 intra-host dataset that increase (A), decrease (B) or don’t alter (C) the tAI of the codon (blue lines). Distributions represent the expected number of synonymous substitutions of each type obtained from random simulations taking into account the trinucleotide mutational flux.

**
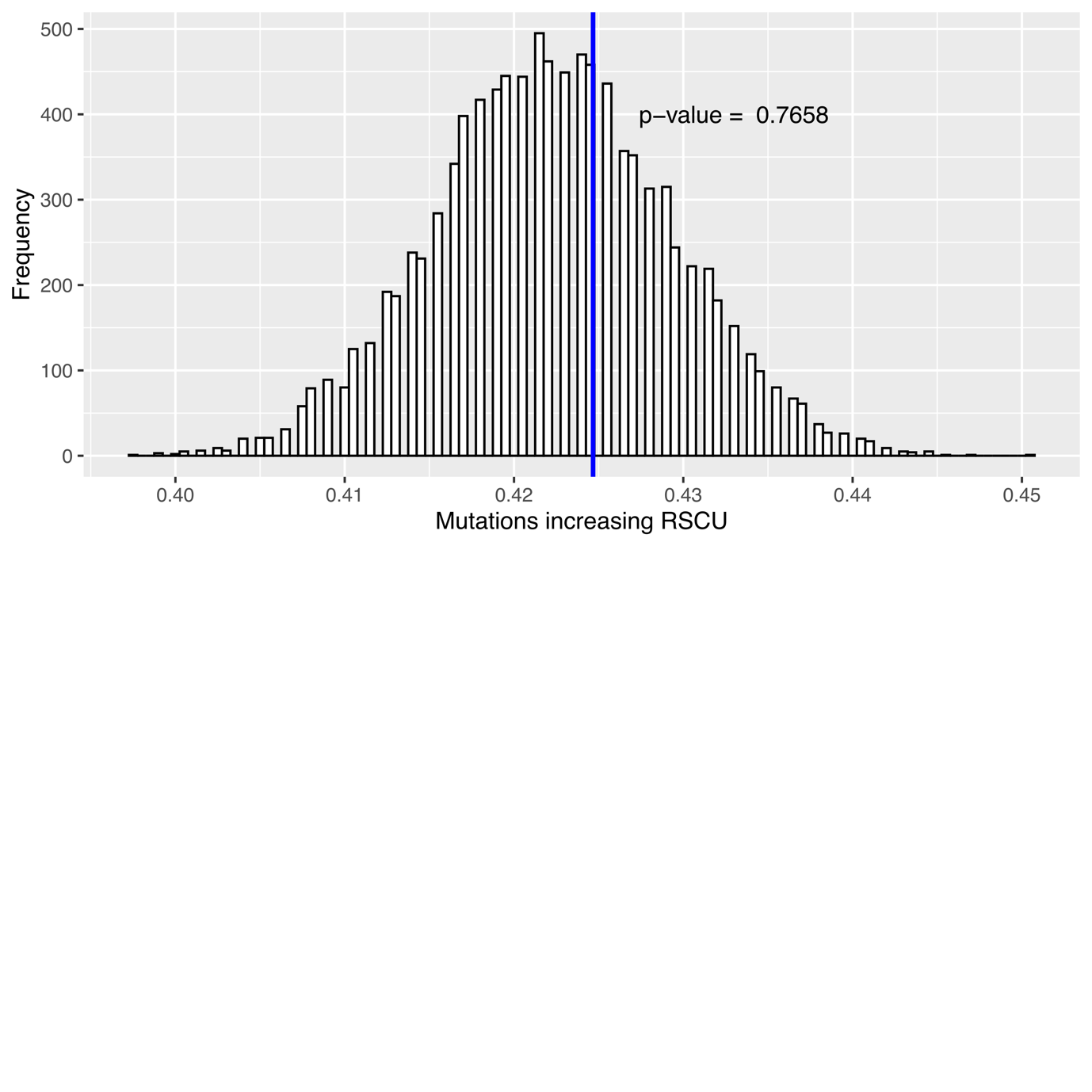
**

**Supplementary Figure 4. 4-fold Mutations in within host SARS-CoV-2 changing Relative Synonymous Codon Usage.** Number of 4-fold synonymous substitutions in the SARS-CoV-2 intra-host dataset that switch to a codon with a higher relative synonymous codon usage in human (blue line). Distribution of expected number of synonymous substitutions that increase RSCU obtained from random simulations taking into account the trinucleotide mutational flux.


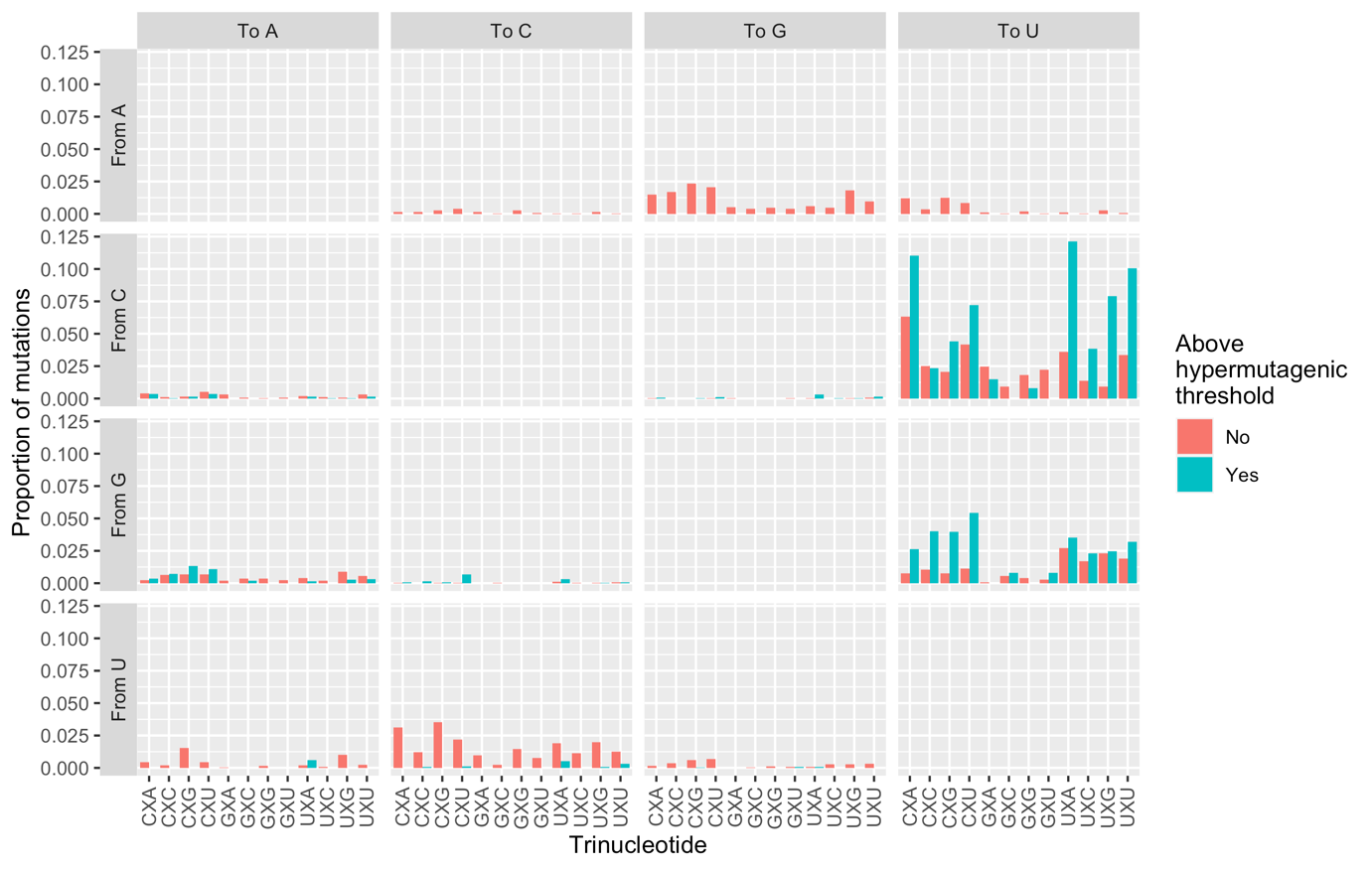


**Supplementary Figure 5. Proportion of four-fold mutations above and below hypermutagenic threshold with trinucleotide context.** Four-fold synonymous mutations in SARS-CoV-2, where mutations are from row nucleotide (left) to column nucleotide (top, e.g. from C to U), and on x-axis trinucleotide context centred on each mutation. Mutations are coloured by whether they occur at a position that is above or below the hypermutagenic threshold, and both groups are scaled to 1 with proportions shown to be more readily comparable.
